# Supplementary material for: Are carbon emissions trading and green financial instruments synergistic? -Comprehensive quantitative research based on content analysis
Source: PLoS One. 2024 Mar 7;19(3):e0298601. doi: 10.1371/journal.pone.0298601 (PMC10919601; doi:10.1371/journal.pone.0298601)
Supplement: S1 Data — (ZIP) [file pone.0298601.s001.zip › Data/地级市Z_F工作报告.zip!/╨┬╜«/┐╦└¡┬Ω╥└╩╨/└┤╫╘╣1⁄2╓┌║┼í╛┬φ┐╦╩2╛▌═°í┐_2018.docx]

各位代表：

现在，我代表市人民政府向大会报告工作，请予审议，并请市政协委员和列席人员提出意见。

2017年工作回顾

一年来，在自治区党委、自治区人民政府和市委的坚强领导下，市人民政府紧密团结和依靠全市各族干部群众，以迎接和学习党的十九大精神为契机，树牢社会稳定和长治久安总目标不动摇，锐意进取、攻坚克难，在社会稳定、经济发展、城市建设、民生改善、生态文明等各方面取得了较好成效，完成了年初确定的目标任务，开创了以稳定成果促发展、以发展成果保稳定的新局面。

2017年全市预计实现地区生产总值720亿元，增长7.5%（按可比价计算，下同），是进入“新常态”以来全市经济发展势头最好的一年。其中，第一产业增加值5亿元，增长2.8%；第二产业增加值498亿元，增长5.3%；第三产业增加值217亿元，增长12.6%。扣除中央石油石化企业贡献份额，实现地方生产总值201亿元，增长8%。实现一般公共预算收入87.5亿元，增长10.5%。全年完成固定资产投资247亿元，增长31.5%。实现社会消费品零售总额66亿元，增长5.5%。居民消费价格上涨0.6%。城镇居民人均可支配收入39000元，增长9%；农牧民人均纯收入22695元，增长9.7%。城镇登记失业率控制在1%以内。

（一）全力落实总目标，社会大局和谐稳定。

（二）持续聚焦改革发展，经济运行稳中有进。我们坚持稳增长、促改革、调结构、防风险协同推进，突出抓好发展质量和效益，经济总体呈现良好发展势头。

新旧动能持续加力。石油石化产业稳健发展，环玛湖区域油田勘探实现新突破，展现了10亿吨级大场面。中石油集团公司出台加快发展新疆油气业务的意见，明确提出实施5000万吨油气当量上产工程，把新疆地区建设成为集团公司稳健发展的主战场、企地和谐发展的示范区，油田可持续发展的基础进一步夯实。独石化公司加工轻烃炼油及乙烯优化调整项目获得中石油集团公司批复。强化为油服务，健全油地协调机制，全力保障石油石化企业加快发展。全年新疆油田公司原油产量1131万吨、天然气28.4亿方，两大炼厂加工原油1290万吨，生产乙烯132万吨。新兴产业发展步伐加快，积极谋划启动了航空物流和临港产业建设，机场改扩建项目即将开工，龙浩集团航空产业平台公司及飞行培训项目注册落地，新开通4条国内航线和克拉玛依—博乐通航航线，机场吞吐量历史性突破39万人次，增长43%。信息产业持续发展，国家“重要基础信息资源战略备份”项目正式落地，影视动漫渲染基地不断壮大，渲染节点数已达4000个，云服务能力显著增强。金融产业实现突破，被国家确定为绿色金融改革试验区。市值上千亿的全疆最大上市公司中油资本和市值数百亿的中油工程落户我市。旅游产业稳步推进，筹划启动荒野特色旅游，以魔鬼城国家5A级旅游景区创建为契机加快推动景区建设，乌尔禾镇成功入选第二批全国特色小镇。全市全年接待游客520.1万人次，同比增长27.8%；国内旅游收入44.5亿元，增长26.4%。

固定资产投资和招商引资全面发力。坚持投资拉动，固定资产投资增速再创新高。招商引资力度空前，出台了招商新政，成立市商务招商局，设立长三角、珠三角、京津冀等招商前线指挥部，建强了招商体系和网络。全年落地项目382个，实现招商引资到位资金160.1亿元，增长78%。

深化改革工作大力推进。继续开展“放管服”改革，进一步减少了审批事项，优化规范了审批流程。“四供”企业分账运行，企业办市政和社会管理等职能移交承接工作步伐加快。推进地方国有企业改革，组建了富城能源集团、油城数据公司，成立了市交投公司、云投公司等投融资平台。激励科技人员创新创业，畅通了科技人员兼职兼薪渠道，先进科技联合研究院、长江大学西部研究院、新油国际院士工作站等创新平台相继落地。8家企业实现重组整合，地方企业力量进一步增强。加快走出去发展，宇飞公司成功落地瓜达尔港、贝肯能源中标乌克兰国家石油公司天然气井钻井项目，地方外向型企业发展质量进一步提高。

发展风险有效管控。加强政府债务管理，规范政府购买服务、政府与社会资本合作，积极化解隐性债务，全市政府债务余额控制在市人大批准的债务限额之内。强化金融监管，认真清理各类交易中心、P2P平台，稳妥处理了一批涉企金融问题，严防发生系统性风险。推动降低金融机构不良率，不良率从6%降到5.16%。

（三）加快推进民生工程，群众幸福感不断增强。我们坚持牢记宗旨、以民为本，始终把群众安危冷暖放在心上，不断保障和改善民生，全年民生支出占公共预算支出的80%，努力让稳定发展成果更多更好地惠及各族群众。

就业创业持续稳定，以创业带动就业，新建了首个创业实训基地，市电子商务孵化基地获批“自治区级创业孵化示范基地”。全年新增创业人数475人，创业带动就业1366人。累计提供就业岗位2.6万个，实现就业再就业3596人。教育惠民扎实推进，全市14934名适龄儿童100%免费就近入园。大力实施民汉“统一编班”，国家通用语言文字授课覆盖率达100%。深化教育“管办评”分离改革，推动学校自主办学，探索实施“中小学校长职级制”。2017年我市高考民汉综合上线率为97.9%。高等教育事业稳步发展，大学城各办学主体招生规模不断扩大，在校学生已达10991人，教学质量持续提升。健康城市建设全面启动，医药卫生体制改革和医联体、医共体建设扎实推进，分级诊疗、家庭签约服务工作取得新成效。成立了市中医医院和市康复医院，中心医院新项目开工建设，形成定位明确、专科特色鲜明的公立医院格局。启动全国疾病诊断收付费改革试点。坚持计划生育基本国策，符合政策生育率达99.1%，违法生育现象得到有效遏制。加强住房保障，完成442套棚户区改造，540套公共租赁住房项目主体封顶。加强物业管理，开展屋面漏水、小区环境治理等专项行动。协助喀什“一市两县”完成阶段性扶贫攻坚任务。以高度的政治责任安置1107名南疆转移就业人员，稳定率达100%。国家公共文化服务体系示范区建设不断巩固提升，成功举办了第九届运动会、“五月中国”艺术展等活动。安全生产形势总体平稳，各项指标均控制在自治区下达的指标范围内。困难人群救助实现全覆盖，残疾人就业渠道不断拓宽，社会养老、福利、救助、慈善等事业取得长足发展。

（四）努力建设高品质城市，美丽克拉玛依建设成效初显。我们坚持规划先行，统筹推进城市建设、精细化管理、生态环境建设和环境污染治理等各项工作，城市品质进一步提升。

城市建设与精细化管理有序推进。坚持规划引领，完成了产业（空间）发展研究，综合交通枢纽规划、航空产业园概念性规划编制取得中期成果，启动城市记忆遗产保护专项规划及代表性项目详细规划。城市建设步伐加快，全年实施民生、基础设施建设项目226项。城市管理水平不断提升，整合设立城市管理委员会，建成了一市四区城市管理信息平台，精细化管理长效运行机制基本建立。违法建筑治理工作有序推进，拆除违法建设面积12.8万平方米。实施小区与公共场所封闭式管理，完成小区封闭224个，公共场所封闭403处，设置防冲撞设施1239处。社会信用体系建设有序推进，信用报告已在政府采购、住房公积金贷款、房产交易中推广使用。文明城市和文明单位创建活动深入开展，城市文明程度和市民素质进一步提高，2017年成功蝉联全国文明城市荣誉称号。

城市生态文明建设持续开展。加大生态系统保护力度，实施了城市周边外围荒漠生态恢复工程，完成造林1.6万亩、封沙育林1.5万亩、荒漠生态建设6003亩。开展玛依湖生态保护恢复工作，启动规划编制以及玛依湖防洪工程项目前期工作。继续实施“大绿化工程”，开展新优植物引种驯化工作，全年新增绿地6710.9亩。积极推进绿色发展，引入共享单车、LNG公交，鼓励市民低碳出行。加大节能减排力度，单位GDP能耗下降10%。推进创建国家级节水型城市。

城市环境污染治理力度空前。以中央环保督察为契机，压实环境保护责任，下大力气解决和治理了一批事关城市发展、市民反映强烈的环境污染问题，持续推动生态环境质量向好发展。深化大气污染防治，实施中心城区供热系统改造一期工程，关停5座燃煤锅炉房、31台燃煤锅炉。推动“奎独乌”大气污染联防联控，进一步削减挥发性有机物排放量。深化水污染防治，全市饮用水源地、河流及水库监测指标达到国家Ⅲ类水质标准，全面实施对金龙湖、玛依湖等5个湖泊的监测和保护。加大土壤污染防治与危险废物监管力度，加快油区危废规范化处置，实施危废闭环监管。

（五）全面加强政府自身建设，行政能力和服务水平不断提升。我们坚持党的领导，努力提升贯彻中央、自治区党委、自治区人民政府和市委决策部署的理解力、落实力、执行力，认真落实向市委请示报告制度，“三重一大”事项提请市委研究决定。扎实推进依法行政，制定政府决策、执法、行政监督等制度16项，系统完备、科学规范的依法行政制度体系初步建成。自觉接受人大的监督，《克拉玛依市城乡规划条例》《克拉玛依市养犬管理条例》已获市人大常委会批准。坚持民主集中制，建立了人大代表、政协委员列席政府常务会议制度，组建了政府法律顾问团，广泛吸纳各方面的意见建议。按照“三个三当”和工匠精神要求，持续转变工作作风，建立了月度经济运行分析、固定资产投资调度等机制。积极向上争取政策、资金、项目支持，一批事关城市长远发展的重大利好得到落实。严明纪律和规矩，实践运用监督执纪“四种形态”，保持惩治腐败高压态势，营造了风清气正的良好政治生态。严格落实中央八项规定，认真查摆“四风”、“四气”问题并积极整改。进一步压缩了一般性财政支出和因公出国计划，努力让有限的财政资金发挥更大效用。持续推进决策、执行、管理、服务和结果公开，保障人民群众知情权、参与权、表达权和监督权。

一年来的稳定发展实践，使我们深切感受到，之所以能够在发展中创新突破，在困难中奋力前行，关键是离不开自治区党委、自治区人民政府和市委的坚强领导，离不开全市各族干部群众紧紧围绕总目标、落实总目标，上下一心、艰苦拼搏！每一位居住、生活在克拉玛依的人，都是这段不平凡历程的亲历者、见证者、推动者！在此，我代表市人民政府，向全市各族干部群众和广大建设者，向市人大代表、市政协委员，向离退休老干部，向各民主党派、工商联、人民团体，向驻市中央企业、兵团团场，向驻市人民解放军、武警部队官兵、公安干警，向所有关心支持克拉玛依稳定发展的社会各界人士，表示衷心的感谢！

各位代表，在总结成绩的同时，我们也清醒的认识到，经济社会发展中仍然存在一些问题和短板，主要表现在：一是经济发展仍不平衡，产业结构尚未发生根本性改变，新兴产业占比相对较小，对地方经济的拉动作用不强，支撑城市转型发展的新业态、新动能还没有完全形成。二是城市精细化管理水平不高，各区公共服务水平不均衡，环保和生态文明建设与各族群众对美丽克拉玛依的需求之间仍有差距，环保工作任重而道远。三是就业、教育、医疗、社会保障等公共服务，与各族群众对幸福生活的向往之间仍有差距。四是政府与市场各就其位、相互促进的关系处理不到位，存在“越位”、“缺位”的问题，“两手合力”作用发挥的不够。下一步，我们将坚持问题导向，直面挑战、敢于担当，着力推动各项问题加快解决。

2018年重点工作安排

2018年是全面学习贯彻党的十九大精神的开局之年，是改革开放40周年，是决胜全面建成小康社会、实施“十三五”规划承上启下的关键一年，是克拉玛依建市60周年的喜庆之年。克拉玛依与全国一道迈入新时代，加速资源型城市转型升级进入关键期、攻坚期。做好今年的工作，责任重大，影响深远。

进入新时代，我们比历史上任何时期都更有信心实现发展目标。这信心源于党的十九大精神和习近平新时代中国特色社会主义思想的正确引领；源于“一带一路”倡议不断深入、“丝绸之路经济带核心区”建设步伐加快带来的重大机遇，中石油集团公司致力推动新疆油气业务大发展带来的重大契机；更源于市委的坚强领导和全市各族干部群众上下一心，众志成城，建设美好家园的磅礴力量和豪迈情怀！

按照中央、自治区党委和市委的要求，结合我市面临的机遇和挑战，2018年政府工作的指导思想是：全面贯彻党的十九大、十九届一中全会、中央经济工作会议精神，以习近平新时代中国特色社会主义思想为指导，深入贯彻习近平总书记系列重要讲话精神、特别是在第二次中央新疆工作座谈会上的重要讲话和视察新疆时的重要讲话精神，紧密团结在以习近平同志为核心的党中央周围，认真贯彻落实自治区第九次党代会，九届一次、二次、三次、四次全会，自治区党委经济工作会议精神和市委对稳定发展改革工作的部署，紧紧围绕社会稳定和长治久安总目标，统筹推进“五位一体”总体布局，协调推进“四个全面”战略布局，牢牢把握稳中求进工作总基调，贯彻新发展理念，加快建设现代化经济体系，围绕人才、资金、信息的支撑，安全、政府软环境、城市硬环境的保障，以及石油石化下游产业链延伸、以油为主的现代服务业、综合交通枢纽的突破口，致力于资源型城市加速转型，努力在石油石化、创新驱动、生态文明和城市建设上寻求新突破，实现大变化；坚持以人民为中心，急群众所急，多谋民生之利、多解民生之忧，在发展中补齐民生短板，保证全市各族群众在共建共享发展中有更多获得感，努力开创克拉玛依稳定发展的新局面。

2018年的预期目标：

——地区生产总值增长7%左右；

——一般公共预算收入增长10%；

——全社会固定资产投资增长15%；

——力争实现招商引资到位资金增长31%；

——社会消费品零售总额增长6%左右；

——居民消费价格涨幅控制在2%以内；

——城镇登记失业率控制在3%以内；

——城镇居民人均可支配收入和农牧民人均纯收入与自治区保持同步增长；

——努力完成节能减排降碳目标任务。

实现上述目标，需要遵循以下基本原则：

一是坚持全面落实市委部署。围绕市委对2018年工作的总体要求，全方位谋划推进各项工作，在理解力、落实力和执行力上下功夫。按照加快建设现代经济体系的要求，围绕推进城市转型和产业升级的支撑、保障和突破口，下大力气，做足文章，力求取得实实在在的成效。

二是坚持稳中求进工作总基调。充分认识“稳”与“进”的辩证统一关系，保持战略定力，尊重经济发展规律，不简单以生产总值增长率论英雄，该稳的要稳住，该进的要进取，全力保持石油石化优势产业稳定发展，加速培育壮大信息、航空物流和临港产业，加快推进“走出去”和外经外贸发展，增强经济韧性和抗风险能力。

三是坚持贯彻落实新发展理念。突出质量第一、效益优先，切实把经济发展的着力点放在调结构转方式上，推动石油石化产业链延伸，加快发展以油为主的现代服务业，大力促进旅游、文化产业创新发展，推动实现更高质量、更可持续的发展。

四是坚持以创新引领发展。把创新作为引领发展的第一动力，以科研、产业创新平台建设为载体，撬动全市创新发展新引擎，加快完善创新体系和制度环境，持续激发市场活力和社会创造力，努力形成人人想创新、人人能创新的生动局面。

五是坚持以人民为中心的发展思想。把以人民为中心的发展思想贯穿在政府各项工作之中，抓住人民群众最关心最直接最现实的危旧房屋修缮、物业服务、就业、医疗、教育等民生问题，既尽力而为、又量力而行，一件事接着一件事办，一年接着一年干，不断增强人民群众的获得感。

在此原则指导下，2018年需要着重抓好以下几方面工作：

（一）以党的十九大精神为引领，聚焦总目标，奋力开创和谐稳定的新局面。习近平总书记在党的十九大报告中指出，“维护国家安全是全国各族人民根本利益所在”，“严密防范和坚决打击各种渗透颠覆破坏活动、暴力恐怖活动、民族分裂活动、宗教极端活动”。

（二）以党的十九大精神为引领，深化供给侧结构性改革，奋力开创改革发展的新局面。习近平总书记在党的十九大报告中指出，“坚持质量第一、效益优先，以供给侧结构性改革为主线，推动经济发展质量变革、效率变革、动力变革，提高全要素生产率，着力加快建设实体经济、科技创新、现代金融、人力资源协同发展的产业体系”。我们要贯彻落实中央经济工作会议精神，深刻把握“我国经济发展已经进入新时代，已由高速增长阶段转向高质量发展阶段”的重大判断，坚持稳中求进总基调，深入贯彻新发展理念，按照高质量发展的要求，以改革创新引领发展，坚持改革不停顿，创新不止步，从财政体制、国有企业、招商体制等方面全力推进深化改革，切实加大简政放权力度，充分调动各区、各企业、各行业的积极性、主动性，转变发展方式、优化经济结构、转换增长动力，使经济更具活力和韧性。

——突出石油石化传统动能的质量变革，在下游产业链延伸和以油为主的现代服务业上实现新突破。

推动石油石化产业链延伸。抢抓中石油集团公司“燃料变原料”的战略调整机遇，全力协调解决独石化公司、克石化公司原油供应配给问题，提高两大炼厂原油加工量。协调国家铁路局支持独石化公司进口哈萨克斯坦轻烃，确保加工轻烃炼油及乙烯优化调整项目原料供应，实现效益最大化。以聚酯和聚乙烯为化工下游产业突破口，重点支持天利实业、天利石化化纤及高端聚乙烯产品发展。支持克石化公司40万吨/年变压器油质量升级、润滑油结构调整、汽柴油国VI标准质量升级等一批重点项目开工建设。

推动以油为主的新兴产业和现代服务业向更高层级迈进。坚持市场配置资源原则，培育一批为油服务克拉玛依品牌，大力推进工程技术服务行业市场化进程，加快领域内企业兼并重组，形成钻井、油田化学下游、科技服务等专业领域的龙头企业，形成具备国际竞争力的大型油气服务集团。补齐装备制造业短板，积极引进机械加工重点企业，带动配套服务，提升要素配置能力。深入推进与哈里伯顿、三一集团、科瑞集团等油田设备制造企业的合作，支持新油国际、宝鸡钢管加快升级发展，培育先进制造业集群。支持本地企业“走出去”，通过联合发展、战略重组、推动上市等方式发展壮大。发挥中国石油大学（北京）克拉玛依校区的作用，培养更多的石油石化专业人才，面向国内外提供更加优质的石油石化工程实训教育。

加快推进涉油总部经济发展。积极争取中石油集团公司新疆区域财务、招标、信息、人力资源、采购等五大共享中心及集团物业服务中心落地我市。推动成立航空煤油销售公司。积极争取中石油等央企参与我市商品交易中心建设，建设服务“一带一路”沿线国家的石油石化产品交易平台。支持中石油光网公司建设运营，推动建成连接国内外的光传输主干网络。

坚持为油服务不动摇。秉承“油地一家人”理念，大力支持协调解决石油石化企业在维稳安保、安全生产、队伍稳定、后勤保障等方面的困难。有序承接企业办市政和社会管理职能，进一步减轻油田企业负担。全力为企业创造良好的发展环境，支持油田持续推进双“6+1”重点工程，争取更多自主经营政策，为油田勘探开发“松绑”。争取2018年新疆油田公司原油产量增加到1145万吨。

——突出创新驱动新动能的动力变革，在增强发展内生动力上争取新转变。

全力推动信息产业发展。围绕提升云服务能力，推动形成丝绸之路经济带重要的信息服务平台，纳入国家大数据（新疆）综合试验区布局。扩大华为、中国移动、中石油等数据中心规模，积极争取中国电信大数据库建设落地，推动直达国内主要城市光纤建设，努力承接更多自治区厅局、周边城市以及中东部地区的数据服务需求，提高云服务辐射能力。进一步做大渲染基地规模，推动产业链向数字影棚、影视制作等全产业生态体系扩展。全力推进互联网、云计算、大数据与实体经济深度融合，加快“两化融合”步伐，支持油城数据公司开展智能化研究与应用，推动地方企业参与油田智能物联网建设。大力发展国际远程医疗、教育课件研发、多语种服务、国际培训等服务外包业务，加快推动大数据在政务服务、平安城市建设、交通、旅游等行业的应用，让信息化建设成果惠民惠企。

增强科技创新带动能力。抢抓丝绸之路经济带创新驱动发展试验区机遇，推动高新区（白碱滩区）争创国家级高新区，力争纳入“国家自主创新示范区”，不断吸引更多的企业、项目落地。加强国内外科研机构、技术转移中心的引进培育，以先进科技联合研究院与上海科技开发交流中心合作项目为突破，不断拓宽在能源化工、智能制造、信息技术、健康医疗等多个领域的科技合作。健全完善“产学研用”机制，鼓励科研院所、高校和企业联合，支持科研团队做大做强。

加快推进深化改革。优化地方国有企业布局，盘活全市各类资产资源，整合优质股权，组建传统产业、新兴产业两大国有资本投资运营公司。推进市燃气公司与昆仑能源公司合作、富城能源集团控股宇澄热力公司，提高资本收益率。推进投融资体制改革，加快绿色金融改革创新试验区建设，建立绿色金融服务优先支持备选项目库，先行安排城市外围荒漠生态恢复与建设、玛依湖保护等项目开展试点。设立支持中小企业发展、促进科学成果转化等产业（股权）投资基金，加大政府与社会资本合作力度。加快金融业态聚集，加强与政策性、开发性金融机构的合作，健全政银企社合作对接机制，拓宽中小企业投融资渠道。深化电力体制改革，推动同网同价，力争两家售配电公司参与市场交易，全力打造全疆低电价区。

集聚招商引资新动能。健全完善投资与贸易促进委员会的功能作用，进一步凝聚招商合力。加大招商引资政策宣传力度，实现人人掌握政策、人人能够招商，形成全民招商的良好氛围。创新招商方式，通过购买服务选择有实力的专业招商中介、银行、咨询公司、律师事务所等，开展委托招商、代理招商，提高招商效率。注重招大引强，围绕石油石化产业、高新科技产业、金融服务产业、文化影视旅游产业、国际贸易物流产业、航空产业等方向，紧盯国家轨道交通综合实验区、魔鬼城影视拍摄基地等一批重点项目，密切跟踪、持续推进、加快落地。实施招商引资项目“保姆”式服务，确保重点项目“招而能落，落而能活”，全年力争实现招商引资到位资金210亿元。

打好防范化解重大风险攻坚战。坚持凡事从最坏处打算，向最好处努力。严守政府债务管理的红线和底线，将自治区发行的政府债券作为唯一举债方式，不以任何方式为任何单位和个人债务提供担保、承诺。规范推进政府和社会资本合作，严守财政承受能力10%红线，依法合规实施政府购买服务，坚决杜绝隐性债务。继续争取自治区置换债券，降低政府债务成本，平滑财政支出。全面加强对小额贷款公司、融资担保公司、融资租赁公司、地方资产管理公司等7类金融机构的日常监管，加大防范非法集资宣传和打击力度，进一步压缩非法集资活动在我市的生存空间，防止发生系统性金融风险。

——突出生态文明新动能的理念变革，在发展绿色经济、低碳经济上谋划新作为。

加快旅游产业发展。深化旅游供给侧结构性改革，启动三平石化特色小镇、独库小镇、克拉玛依航空小镇等建设，加快发展乌尔禾特色小镇、玛依湖保护等特色旅游项目。推动“旅游+通用航空、汽车服务、文化体育、智慧旅游、工业农业”等产业融合发展，努力实现世界魔鬼城创建国家5A级旅游景区的目标，不断丰富旅游业发展新业态和旅游市场有效供给。完善旅游基础设施和产业要素建设，大力发展具有克拉玛依特色的民宿业和农业体验旅游，全面提升旅游服务质量和管理水平，不断满足人民日益增长的美好生活需要。围绕“荒野之旅”“时尚之城”，加大城市精准营销力度，提升城市旅游品牌的美誉度和知名度。推动旅游业成为引领我市经济转型升级的新引擎，完成“首批国家级旅游业改革创新先行区”三年期任务，全市旅游接待、旅游收入增长25%以上。

推动文化产业创新发展。建设影视动漫渲染基地，支持中小微等各类影视企业发展，形成全产业链业态。启动克拉玛依音乐主题场馆建设，推动民族音乐展演、丝路文化交流、音乐作品孵化与发行。坚持以“文化创意+”为引领，依托文化创意产业园，培育一批创意骨干企业和品牌，推动创意经济加快发展。借力新闻出版广电改革，组建文化企业集团，推出一批以传播石油文化为主要内容的影视、出版、演艺等精品工程，推动优秀文化走出去，让石油文化和本地文化薪火相传、发扬光大。

推进现代农业建设。发挥现代农业园区示范带动作用，统筹农业基础设施建设，大力培育家庭农场、农业生态观光园等新型农业经营主体。实施5000亩绿色蔬菜瓜果基地建设。持续推进草原畜牧业转型，推动养殖方式由传统放牧向舍饲、半舍饲等方式转变，提高畜牧业产出率。加大“三品一标”认证和标准化生产力度，推进多种形式适度规模经营。推动牲畜品种改良，引导精品肉牛、肉羊规模化养殖，实现畜产品多样化。推进土地确权登记颁证试点，深化农村土地三权分置改革和产权制度改革，壮大集体经济。

争创国家生态园林城市。践行“绿色”发展和“绿水青山就是金山银山”的理念，按照到2020年成功创建国家生态园林城市的目标，有序推进各项工作。加大本地苗木培育力度，实施会展中心带状公园、独山子城市公园等41项绿化工程，完成新增绿地面积6042亩。持续推进环城生态圈建设，启动编制荒漠生态建设实施方案，完成人工造林1.5万亩，封沙育林2万亩。加大玛依湖、古海湿地保护利用力度，依托湖泊、湿地、水系资源，努力将我市建设成为戈壁水乡。

打好污染防治攻坚战。严守“三大底线”，全力构建政府为主导、企业为主体、社会组织和公众共同参与的环境治理体系。全面实施新一轮大气污染防治工作，大力推进重点石化企业环保提标升级改造，实现独山子区挥发性有机物排放总量在2016年基础上削减20%以上，关停或拆除中心城区所有燃煤供热锅炉房，增强群众的蓝天幸福感。落实土壤污染防治方案，督促规范化处置历史遗留含油污泥。启动艾里克湖治理工程，全力推进白杨河—艾里克湖流域水污染防治，促进艾里克湖水质逐年提升。

——突出对外开放发展，在融入“丝绸之路经济带核心区”发展中实现新布局。

全力加快综合交通枢纽建设。积极发展航空物流和临港产业，编制完成综合交通枢纽等规划，加速推进克拉玛依机场改扩建工程和口岸机场报批，积极争取航空公司在克拉玛依设立基地。积极申办全国民航航班时刻协调会，开通更多通往内地重点城市、疆内旅游城市和景区的直飞、串飞、环飞航线，加大空地联运工作力度，全年力争机场客运吞吐量突破50万人次。支持龙浩飞行培训学校发展，推进乌尔禾通用航空机场建设，申报国家通用航空产业发展示范区。推动公路建设，继续开展省道201两侧改造工程、胜利路南延等道路项目建设，打通我市对外交通大通道。推动铁路建设，完成克拉玛依—奎屯城际铁路提速工程的前期工作。

加快“走出去”发展步伐。秉持“开放”发展理念，以服务“一带一路”建设为导向，积极参与中巴、中蒙俄等经济走廊建设，搭建外经贸公共服务平台，提升服务便利化水平，全年力争实现外贸进出口总额8000万美元。支持工程承包、油气技术服务、油气装备等优势领域企业扩展海外业务，深化与周边国家在能源开采、装备制造等领域的合作，带动我市产品和服务出口。积极谋划与瓜达尔区的合作，主动参与瓜达尔园区建设。拓展免税（保税）直营店业态，培育消费新热点。加强沪克全面合作，与科研机构、大学等建立合作关系，依托政策、资金、市场等借力发力、联动发展。加强兵地融合发展示范区建设，深化与兵团七师、十师的全面融合发展，在维稳、产业发展、旅游开发等方面与塔城地区建立全方位融合发展格局。

——突出人才人口发展，在引进高端人才、增加城市人气上实现新思维。

坚持人才强市，以广纳天下人才的胸怀、容才用才的气度，真正把人才引进来、留得住、用得好，为全市经济社会全面发展提供坚强的人才支撑。利用1+10系列人才新政，全面落实“1150”高层次人才引进工程，加强上海专家博士后与我市企事业单位的项目合作，共建专家服务基地。推进自治区博士后创新实践基地建设，落实“油城英才·高层次人才”特殊支持计划，做好领军拔尖人才的培养、管理、服务。探索实施高技能人才多元化评价机制，开展首席技师的选拔培养，推进技能大师工作室建设，拓宽技能人才晋升通道。依托国内知名专业人力资源服务机构，搭建我市人力资源服务平台。优化城市户籍管理，为专业技术人才、外来劳动力等城市急需人口建立绿色通道，推进多层次住房供给体系建设，进一步降低外来人口生活成本，吸引来克创业就业。发挥企业引进外来劳动力的主体作用和积极性。制定优惠政策，鼓励人才更多的聚集在基层一线发挥作用。

（三）以党的十九大精神为引领，推进区域协调发展，奋力开创城市建设管理的新局面。习近平总书记在党的十九大报告中指出，“坚定走生产发展、生活富裕、生态良好的文明发展道路，建设美丽中国，为人民创造良好生产生活环境”。我们要坚持人民城市为人民，以健全完善全国文明城市创建长效机制为抓手，推动城市建设精品化与管理水平精细化，提升市民文化素养，让城市更有品质，更有温情，让各族群众在城市生活得更方便、更舒心、更美好。

全面开展“城市双修”。强化规划引领，结合生态资源修复和城市功能修补开展相关规划编制。做好机械制造总公司、物资供应总公司厂区保护，启动实施黑油山片区石油工业记忆工程、老粮食局片区城市发展记忆工程。强化物业管理，建立健全四级共管的物业服务监管体系。全面提升夜间景观品质，对准噶尔路、南新路、友谊路等城市重要轴线和节点开展景观亮化。持续优化城市绿化美化品质，突破惯性绿化观念和手法，丰富绿化植物种类，增加花卉和爬藤类植物种植量，实施带状绿化、街头绿化、观光花海和胜利路南延荒漠生态绿化工程，形成“三季有花，四季有景”的景观效果。

实施精品建设和精细管理。扎实推进重大项目建设，形成规划一批、实施一批、论证一批、储备一批的良性循环。全力推进中心医院新项目、工程教育基地、实验检测中心等工程压茬进行。加快保障性住房建设，实施独山子区南园小区等8个棚改项目和克拉玛依区佳福小区（三期）等6个公租房项目。推动城市精细化管理“大联动”机制常态化、规范化运行，推进“我的克拉玛依”市民APP建设，广泛调动市民力量参与网格化服务管理。加快诚信体系建设，拓展信用报告应用领域。持续挖掘克拉玛依匠心故事，彰显工匠文化，传承石油基因。巩固提升国家公共文化服务体系示范区建设，推进数字文化馆、数字图书馆建设，广泛开展高质量文化活动，增强市民对文化服务的获得感。

（四）以党的十九大精神为引领，不忘初心，奋力开创民生改善的新局面。习近平总书记在党的十九大报告中指出，“增进民生福祉是发展的根本目的”。保障和改善民生没有终点站只有新起点，我们要扭住惠民工程，提高保障和改善民生水平，不断在幼有所育、学有所教、劳有所得、病有所医、老有所养、弱有所扶上取得新进展，让克拉玛依新时代的发展列车承载起每一个人的梦想，让各族群众的生活一年更比一年好，不断增强获得感和幸福感。

坚持就业优先。重点抓好未就业高校毕业生就业，实施就业困难人员“一对一”帮扶和“零就业家庭”动态清零。推动创业带动就业，进一步优化大众创业万众创新的政策环境，继续抓好创业孵化基地建设，大力开展创业实训和创业指导，2018年发布就业岗位5000个以上。

大力发展教育事业。继续支持中国石油大学（北京）克拉玛依校区的建设和发展，扩大厚博学院、克拉玛依职业技术学院的招生规模及社会影响力，努力建成集教学、科研、社会服务、文化引领于一体的工程教育基地。鼓励和引进国内各类知名学校在克拉玛依设立分校或联合办学，积极与上海对外开放大学对接，建设克拉玛依工匠学院，集成市民学校、健康学校、电视大学、老年大学的资源，形成“规划统一、师资共享、课程多元、线上线下融合”的办学模式，力争成立克拉玛依城市大学。深化教育管办评分离改革，实施并完善中小学校长职级制，落实学校办学自主权，不断完善学校内部治理结构。持续加强中小学思想政治工作，开展爱国主义教育、理想信念教育、民族团结教育。持续实施未来教育家培养工程和卓越教师培养计划，建设一流的教育人才队伍，不断提升教育治理体系和治理能力现代化水平，加快推进教育现代化。

扎实推进健康克拉玛依建设。着力构建健康环境、健康社会、健康产业和健康人群“四大工程”，深入开展健康细胞工程建设。持续深化医药卫生体制改革，加快建立管办分开、政事分开的现代医院管理机制，实施公立医院去行政化管理。加快医共体、专科联盟等医联体建设，深入实施公立医院差异化发展，做优、做强三级综合医院，做专、做精二级医院，做实家庭签约服务。与社会资本合作成立妇幼医院，成立精神病院、传染病院，鼓励非公立医院专科化、差异化、规模化和高端化发展，满足多元化医疗服务需求。利用远程医学平台优势，进一步加强与四地五师及周边医疗机构合作，逐步建成辐射周边的医疗服务中心。全面从严控制违法生育，降低并稳定适度生育水平，促进人口均衡发展。

加强社会保障体系建设。全面启动长期护理保险，实施城乡居民大病医疗保险、人身意外伤害保险等制度，进一步完善多层次的医疗保障体系。积极推动全国疾病诊断收付费改革试点，促进医保基金支付更加合理。加大疆内异地就医结算和跨省异地就医结算工作力度。稳步推进全民参保登记计划，促进社会保障由制度全覆盖向法定人员全覆盖转变。推进建筑业参加工伤保险“同舟计划”，深入推进工伤预防试点，进一步扩大社会保险覆盖范围。

深化暖心工程。将危旧房屋修缮作为2018年第一民生工程，全面推动老旧小区的治理改造。持续投建绿色蔬菜平价直销店，保持“菜篮子”价格平稳。积极发展养老产业，推动新概念养老公寓、中益克拉玛依生态养生养老示范基地建设。在产业培育、就业创业、医疗教育、旅游文化、人才培养等方面对喀什“一市两县”开展全方位的帮扶，巩固扶贫成效，提高扶贫质量，确保完成阶段性扶贫攻坚任务。

持续推动安全生产。始终把人的生命安全放在首位，持续推进安全生产领域改革发展，强化属地监管和企业主体责任，形成全行业督导和协调的综合监管格局。加大高危重点行业领域整治力度，对非法生产经营建设活动顶格处理，绝不姑息，坚决遏制重特大事故发生。加快推进市、区两级应急联动信息平台建设，完善各项应急预案、救援救助机制，提升突发事件应急处置能力和保障水平。加强食品安全溯源体系建设，推广“阳光厨房”，让老百姓吃得放心。

（五）以党的十九大精神为引领，弘扬主旋律，奋力开创民族团结宗教和谐新局面。习近平总书记在党的十九大报告中指出，“深化民族团结进步教育，铸牢中华民族共同体意识，加强各民族交往交流交融，促进各民族像石榴籽一样紧紧抱在一起，共同团结奋斗、共同繁荣发展”。

奏响民族团结新乐章。始终高举各民族大团结旗帜，传承发扬克拉玛依与生俱来的民族团结基因，进一步加强各民族交往交流交融，形成你中有我、我中有你、密不可分的崭新局面。大力挖掘和选树民族团结进步先进典型，在全社会积极营造共居共学共事共乐的浓厚氛围，让民族团结基因一代一代传下去。全市总动员，全面开展民族团结宣传教育活动，广泛深入推进全国民族团结进步示范城市创建工作，形成全民受教育、人人促团结的生动局面。

（六）以党的十九大精神为引领，厘清政府与市场关系，奋力开创政府自身建设的新局面。习近平总书记在党的十九大报告中指出，“转变政府职能，深化简政放权，创新监管方式，增强政府公信力和执行力”。我们要坚决贯彻中央、自治区党委、政府和市委的决策部署，牢固树立“四个意识”、“四个自信”、“四个服从”，坚持党的领导，坚持以人民为中心，树立正确的政绩观，保持“赶考”姿态，坚定“考好”决心，以即将开展的“不忘初心、牢记使命”专题教育活动为契机，持续加大对党的十九大精神的学习领会，持续提升政府治理体系和治理能力的现代化，着力打造“五型政府”，力争向全市人民交上满意答卷。

着力打造“诚信型政府”。按照习近平总书记“讲诚信、懂规矩、守纪律”的要求，自觉用习近平新时代中国特色社会主义思想武装头脑、指导实践，持续增强政治自觉、思想自觉和行动自觉，坚持知行合一，各级领导干部带头忠诚守信、遵守规矩、筑牢底线，使讲诚信在政府系统蔚然成风。深入推进依法行政，坚持法定职责必须为、法无授权不可为，强化对行政权力的监督制约，坚持在市委领导下，自觉接受人大监督和政协民主监督，认真听取民主党派、工商联及各人民团体意见，让权力在阳光下运行。积极兑现对社会、对企业、对群众的承诺，做到言必信、行必果，不断提高政府公信力。

着力打造“高效型政府”。正确处理政府和市场的关系，放开市场这只“看不见的手”，发挥市场在资源配置中的决定性作用，用好政府这只“看得见的手”，进一步提高宏观调控水平，两手合力，形成市场作用和政府作用有机统一、相互补充、相互协调、相互促进的格局，推动经济社会持续健康发展。持续推进简政放权，厘清市、区两级政府的权责关系，按照“人随事走、编随人走、财权匹配”的原则，逐步推进人员编制、财力等资源向区级下沉，重点扩大区级政府在投资、城市建设管理、行政执法、市场监管等领域事权，提高各区干事创业的积极性。持续深化审批制度改革，推行政府系统限时办结制，审批部门向服务窗口充分授权，推动政务大厅办事和网上系统一体化建设，让信息多跑路、群众少跑腿，使政务服务中心成为真正的“审批”中心。进一步改进作风、狠抓执行、提升效能，形成抓落实的强大合力，确保自治区和市委各项决策部署落地生根。

着力打造“服务型政府”。坚持“把困难留在机关，把方便留给基层和群众”，凡事想着群众，工作依靠群众，在人性化管理中提高服务水平，下大力气解决危旧房屋修缮、物业服务管理、养老医疗等群众关心关注的民生问题，让群众享受到优质的公共产品和服务。全力以赴为企业服务，抓住克拉玛依特殊的资源优势、特殊的文化底蕴、特殊的地形地貌，在“特”字上做文章，创新招商引资模式，优化营商环境，吸引更多企业到克拉玛依投资兴业。

着力打造“开放型政府”。进一步解放思想，放开思路，拓宽视野，围绕克拉玛依的可持续发展和产业转型升级，去除制约开放发展的各种体制机制障碍，突出问题导向找方法，突出发展导向找思路，突出特色优势找路径，突出借智发展找载体，努力在持续解放思想中深化改革开放，实现发展目标。主动将城市转型融入“一带一路”、“丝绸之路经济带核心区”建设等国家和自治区发展战略上来，找准克拉玛依在国家和自治区发展战略中的角色定位，以“十三五”规划中期调整为契机，扩大厅市合作范围，加大到自治区各委办厅局的沟通联系力度，紧扣自治区“丝绸之路经济带核心区”五大中心规划，大力推动重点项目进入自治区相关规划，争取政策资金项目支持。

着力打造“廉洁型政府”。严格执行重大事项决策、重要人事任免、重大项目安排和大额度资金使用等“三重一大”事项集体决策制度。加强财政收支管理，全面实施绩效管理，增强可持续性，牢固树立过“紧日子”的思想，坚持尽力而为、量力而行，严格财政支出审批监管程序。强化政府监管，深度治理违法违规行为，着力打造公平公正的建筑领域市场秩序。严格落实中央八项规定实施细则、自治区党委和市委关于作风建设的规定，进一步压缩一般性支出，严格控制“三公经费”，确保有限的财力用在关键领域。深入开展党风廉政教育，落实党风廉政建设责任制、廉政谈话等制度，建立领导干部重要情况报告制度，加大政务监督、行政监察和审计监督力度，强化对行政权力的制约，扎紧制度的笼子。

各位代表，历史只会眷顾坚定者、奋进者、搏击者，而不会等待犹豫者、懈怠者、畏难者。此时此刻，我们迎来了国家“一带一路”伟大倡议深入推进的历史机遇期，迎来了建市60周年的甲子华诞，放眼展望美好明天，我们倍感幸运，满怀豪情。让我们更加紧密地团结在以习近平同志为核心的党中央周围，在自治区党委、自治区人民政府和市委的坚强领导下，按照新时代提高新站位、新时代贯彻新思想、新时代牢记新使命、新时代展现新作为、新时代书写新篇章的要求，以永不懈怠的精神，锐意进取、埋头苦干，为建设团结和谐、繁荣富裕、文明进步、安居乐业的克拉玛依而努力奋斗！
